# The SAV1322 gene from *Staphylococcus aureus*: genomic and proteomic approaches to identification and characterization of gene function

**DOI:** 10.1186/s12866-016-0824-2

**Published:** 2016-09-06

**Authors:** Jung Wook Kim, Hyun-Kyung Kim, Gi Su Kang, Il-Hwan Kim, Hwa Su Kim, Yeong Seon Lee, Jae Il Yoo

**Affiliations:** 1 Division of Antimicrobial Resistance, Center for Infectious Diseases, National Research Institute of Health, Centers for Disease Control and Prevention, Cheongju, South Korea; 2 Korea National Research Institute of Health, Osong Health Technology Administration Complex, 187, Osongsaengmyeong2-ro, Osong-eup, Heungdeok-gu, Cheongju-si, Chungcheongbuk-do 363-700 South Korea

**Keywords:** Heat shock proteins, Microarray analysis, Microbial drug resistance, Physiological stress, Proteomics, *Staphylococcus aureus*

## Abstract

**Background:**

Bacterial two-component regulatory systems (TCRS) are associated with the expression of virulence factors and antibiotic susceptibility. In *Staphylococcus aureus*, 16 TCRS types have been identified. The histidine kinase/response regulator SAV1321/SAV1322 in the *S. aureus* shares considerable homology with the TCRS DesKR in *Bacillus subtilis*. However, a function for the SAV1322 locus has not yet been assigned.

**Results:**

Deletion of the SAV1322 locus in *S. aureus* results in reduced growth when cultured under low (25 °C) and high (46 °C) temperature conditions. The *sav1322* deletion mutant is more tolerant to oxidative stress in vitro and is less pathogenic in a murine infection model when compared with wild-type parent strain Mu50. Furthermore, the *sav1322* mutant exhibits lower MICs for gentimicin, tetracyclines and glycopeptides, increased autolysis, and a thinner cell wall when compared with the wild-type strain. Microarray and proteomic analyses show that the expression of cell-wall-associated genes *glmS* and *murZ* are lower, and the expression of heat shock and stress-related genes (*hrcA*, *ctsR*, *dnaK*, *dnaJ*, *grpE*, *clpB*, and *clpC*) are higher in the *sav1322* mutant when compared with the wild-type strain. In addition, the *sav1322* mutant displays altered expression of proteins involved in carbohydrate/energy metabolism, cell wall metabolism, and stress or heat shock response, as well as other metabolic processes including lipid metabolism, amino acid biosynthesis, purine or pyrimidine metabolism, transcription, and protein biosynthesis.

**Conclusions:**

The *S. aureus* SAV1322 locus plays a pronounced role in temperature adaptation, antibiotic resistance, and virulence by regulating a wide range of genes and proteins involved in metabolism and stress tolerance.

**Electronic supplementary material:**

The online version of this article (doi:10.1186/s12866-016-0824-2) contains supplementary material, which is available to authorized users.

## Background

*Staphylococcus aureus* is a major cause of nosocomial infections, resulting in increased morbidity and mortality worldwide. Commonly reported methicillin-resistant *S. aureus* (MRSA) exhibits resistance to multiple chemotherapeutic agents, including β-lactams, quinolones, and aminoglycosides, while community-acquired MRSA is associated with serious infectious disease, sepsis, and pneumonia [1, 2]. More recently, vancomycin-intermediate or vancomycin-resistant *S. aureus* (VISA or VRSA) strains have emerged in several countries. These factors have rendered successful treatment of these infections increasingly problematic.

*S. aureus* has survived and thrived over the years in part because of its adaptability and stress response capabilities. It is resistant to a variety of environmental factors, including oxidative, pH, osmotic, antibiotic and temperature stressors [3]. Two-component regulatory systems (TCRSs) act in response to a stimulus that allows cells to sense and respond to changes in many different environmental conditions. Most *S. aureus* strains are endowed with 16 sets of genes that encode TCRSs. An additional TCRS is present in the staphylococcal cassette chromosome mec in MRSA, and this is linked to the induction of methicillin resistance [4]. The well-studied TCRS Agr is a positive regulator of exoproteins, including proteases, hemolysins, and toxins [5, 6]. Other TCRSs, such as SaeSR and ArlSR, influence the expression of some virulence factors [7, 8]. Another system, SrrAB, is homologous to the *Bacillus subtilis* TCRS ResDE; it is involved the modulation of anaerobic gene expression and sensitivity to oxygen tension [9]. The *S. aureus* TCRS VraSR is homologous to *B. subtilis* YvqEC, and it modulates cell wall biosynthesis and increased resistance to vancomycin [10]. Another *S. aureus* TCRS, NreBC, controls nitrate reductase and nitrite reductase operons [11]. More recently, it was reported that the *S. aureus* TCRS WalKR is involved in cell wall metabolism, cell survival, and vancomycin resistance [12, 13].

Although most TCRSs identified in *S. aureus* have been well studied, the functions of a few remain elusive or only partially explained. Of these, the uncharacterized SAV1321/SAV1322 (Mu50) TCRS demonstrates homology with *B. subtilis* DesKR. DesKR has been described as a regulatory system involved in the maintenance of membrane fluidity in response to temperature downshift. With decreasing temperature, the membrane-bound sensor kinase DesK phosphorylates its corresponding response regulator, DesR, which then binds to a specific recognition sequence in the promoter region of the *des* gene to activate its transcription [14]. Finally, activity of the membrane-located fatty acid desaturase Des maintains membrane fluidity in the cold [15].

In this study, we generated an *S. aureus* mutant lacking the SAV1322 locus to examine its role in bacterial physiology and virulence. Comparative microarray and proteomic analyses were performed to determine putative members of the SAV1322 regulon.

## Results

### Construction and characterization of the S. aureus sav1322 knockout mutant

**Fig. 1:**
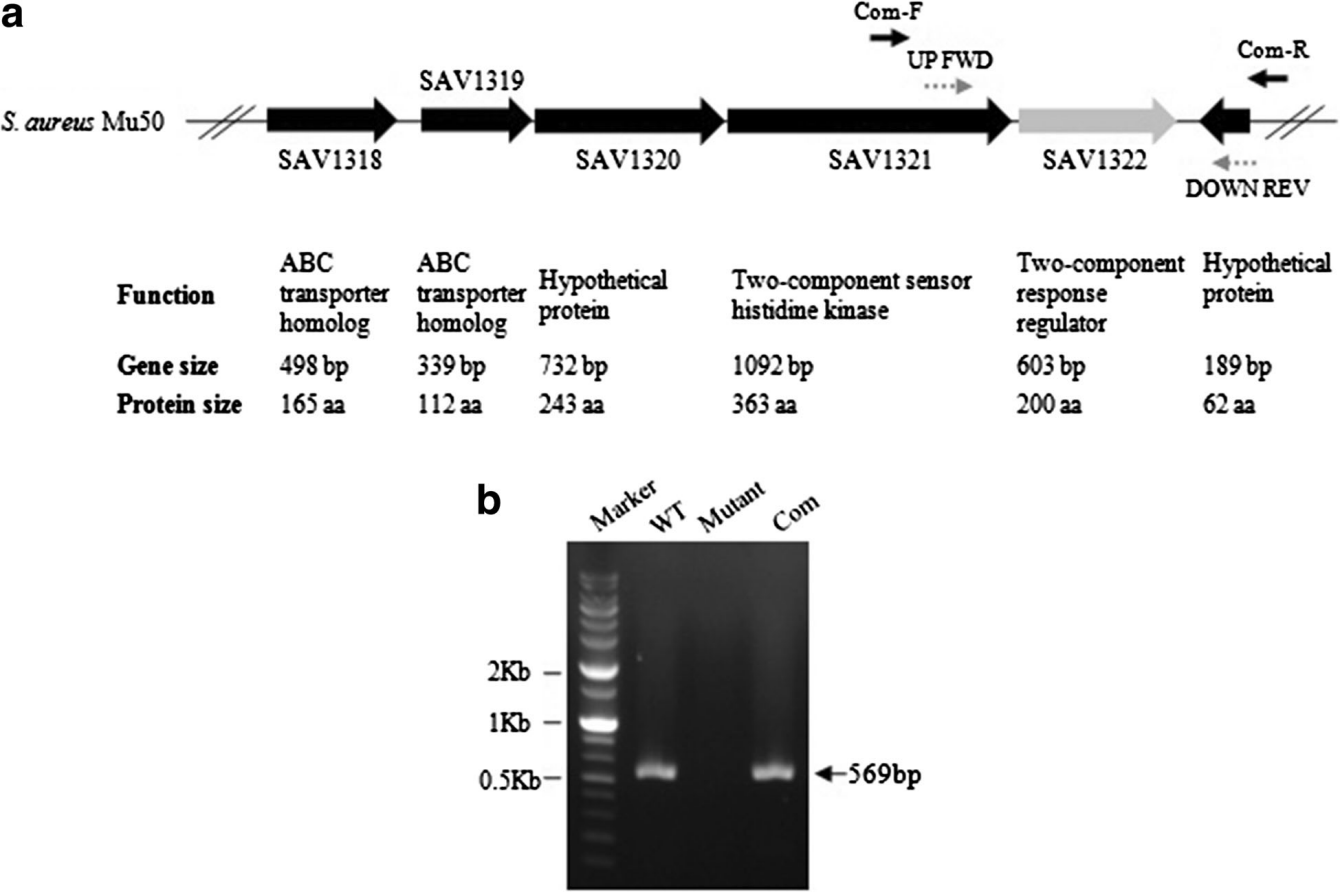
**a** Genetic structure of the SAV1322 locus in the wild-type (WT) *S. aureus* Mu50 strain. **b** RT-PCR analysis of the *sav1322* mutant

A *sav1322* knockout mutant strain was created from the wild-type (WT) *S. aureus* Mu50 using homologous recombination. Re-introducing the SAV1322 locus into the *sav1322* mutant generated a complementation strain. The WT SAV1322 locus is shown in Fig. 1a. To confirm allelic replacement, chromosomal DNA was isolated from the WT, *sav1322* mutant, and complementation strains. PCR analysis, sequencing, and RT-PCR confirmed the mutation (Fig. 1b).

To determine whether deletion of the *S. aureus* SAV1322 locus had any impact on bacterial growth and morphology, growth curves and colony forming units (CFU) were compared at 37 °C. We observed no difference in growth between the WT and the *sav1322* mutant strains at this temperature. Similarly, no obvious differences in colony morphology were observed (data not shown).

### Temperature stress

**Fig. 2:**
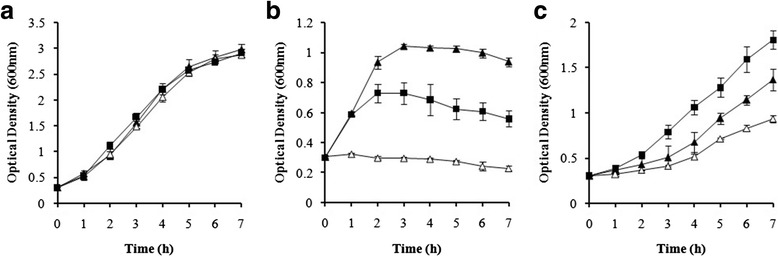
Growth rates of the *S. aureus* Mu50 (WT, filled triangle), *sav1322* mutant (open triangle), and complementation (Com, filled square) strains at (**a**) 37 °C, (**b**) 46 °C, and (**c**) 25 °C. Data are expressed as mean ± SD values for the measurements of absorbance in 600 nm

To examine the role of the SAV1322 locus in response to heat and cold stress, we compared the growth rates of the WT, *sav1322* mutant, and complementation strains at 25 and 46 °C (Fig. 2). The *sav1322* mutant grew less at 46 °C when compared with the WT strain. The WT strain exhibited a normal growth curve from inoculation until 3 h, at which point a decline in growth was noted. The complementation strain exhibited a growth curve that was similar to the WT; however, a growth decline was noted after 1 h (Fig. 2a). At 25 °C, growth of the WT and mutant strains started to increase at 3 h, yet the growth rate for the WT strain was faster than that of the *sav1322* mutant strain. Growth of the complementation strain increased continuously, beginning immediately after inoculation at 25 °C (Fig. 2b). To determine growth arrest or viability in temperature shifts, we measured the CFU of strains every hour after inoculation (Additional file 1). At 46 °C, mutant strain maintained the CFU from 1 h to 3 h and declined the viability after 4 h. At 25 °C, all strains maintained the CFU during the measurement (7 h). It is suggested that mutant strains exhibited growth arrest in early phase and after declining viability.

### Oxidative stress

**Fig. 3:**
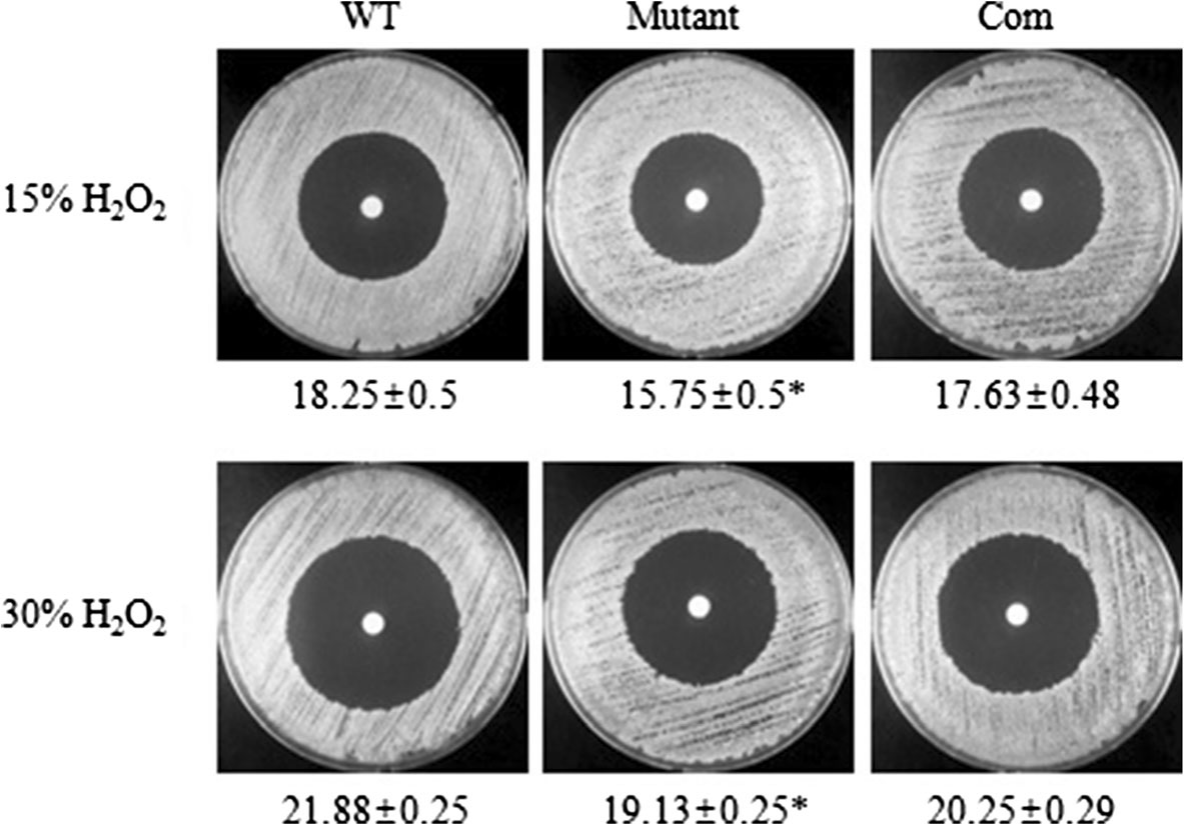
Oxidative stress response of the wild-type (WT) *S. aureus* Mu50, *sav1322* mutant, and complementation strains (Com). Data are expressed as mean ± SD values for the zone of inhibition diameter (mm). Asterisks denote statistical significance (*p* < 0.001) when compared with the WT strain

To examine the role of the SAV1322 locus during oxidative stress, a disk diffusion assay with two different concentrations of hydrogen peroxide (H_2_O_2_; 15 and 30 %) was performed. When compared with the WT strain, the *sav1322* mutant strain displayed greater resistance to both hydrogen peroxide concentrations tested (Fig. 3). Sensitivity of the complementation strain to oxidative stress was similar to that of the WT strain for both hydrogen peroxide concentrations tested (Fig. 3).

### Microarray transcriptional analysis

All of the microarray data have been deposited in the NCBI’s Gene Expression Omnibus (GEO) under accession number GSE85824. A comparison of the Mu50 and the *sav1322* mutant transcription profiles revealed differential expression of 17 genes (Table 1). Notably, expression of two genes associated with cell wall peptidoglycan synthesis (*murZ*, encodes UDP-N-acetylglucosamine 1-1carboxylvinyl transferase and *glmS*, encodes glucosamine-fructose-6-phosphate aminotransferase), was lower in the *sav1322* mutant strain. Conversely, transcription of the chaperone genes *dnaJ*, *dnaK*, and *grpE*, as well as the negative heat shock regulator genes, *hrcA* and *ctsR*, was higher in the *sav1322* mutant strain. Furthermore, transcript levels of two genes encoding Clp ATPases, *clpB* and *clpC*, were higher in the *sav1322* mutant strain.

**Table 1:** Representative genes differentially expressed in the WT and *sav1322* mutant strains

Gene locus	Gene	Description	Function	Fold change in *sav1322* mutant
SAV1147	*sdhC*	Succinate dehydrogenase cytochrome b-558		−1.65
SAV2123		Hypothetical protein		−1.64
SAV2124	*murZ*	UDP-N-acetylglucosamine 1-carboxylvinyl transferase	Cell wall biogenesis	−2.02
SAV2125	*glmS*	Glucosamine-fructose-6-phosphate aminotransferase	Cell wall biogenesis	−1.64
SAV0052	*ermA*	Adenine N-6-methyltransferase		1.55
SAV0522	*ctsR*	Transcriptional regulator CtsR	Stress response Transcription regulation	1.76
SAV0523		Hypothetical protein		2.01
SAV0524		ATP: guanido-phosphotransferase		1.92
SAV0525	*clpC*	ATP-dependent Clp protease ATP-binding subunit ClpC	Stress response	1.72
SAV0975	*clpB*	ClpB chaperone homologue	Stress response	2.13
SAV1350	*citB*	Aconitate hydratase	Tricarboxylic acid cycle	1.55
SAV1579	*dnaJ*	Chaperone protein DnaJ	Stress response	1.56
SAV1580	*dnaK*	Chaperone protein DnaK	Stress response	2.30
SAV1581	*grpE*	Heat shock protein GrpE	Stress response	3.96
SAV1582	*hrcA*	Heat-inducible transcriptional repressor	Stress response	3.16
SAV1799		Hypothetical protein		2.23
SAV2497		Putative membrane protein		1.82

### Two-dimensional gel electrophoresis of the WT and sav1322 mutant strains

Two-dimensional gel electrophoresis was used to examine differences in protein profiles between WT and *sav1322* cell lysates (Additional file 2). We observed that proteins associated with carbohydrate metabolism, including those involved in the tricarboxylic acid (TCA) cycle exhibit lower expression in the *sav1322* mutant when compared with the WT strain (Table 2). Moreover, several proteins involved in glycolysis/gluconeogenesis were differentially expressed; expression of fructose-biphosphate aldolase (FbaA) and alcohol dehydrogenase (Adh) proteins was greater in the *sav1322* mutant, while expression of transketolase, phosphoglyceromutase (GpmA), and pyruvate kinase (PykA) was lower when compared with the WT strain. We also observed a lower expression levels for L-lactate dehydrogenase (LctE) and higher expression of D-lactate dehydrogenase (Ddh) in the mutant; both of these proteins are associated with the interconversion of pyruvate and lactate.

**Table 2:** Representative proteins differentially expressed in the WT and *sav1322* mutant strains

Spot ID	Accession^a^	Protein name	Protein Score	Gene	Locus^b^	Expression change
Carbohydrate metabolism and transport						
A023	gi|15924402	Dihydrolipoamide succinyltransferase	714	*odhB*	SAV1412	Down
A025	gi|15924086	Dihydrolipoamide dehydrogenase	1153	*pdhD*	SAV1096	Down
A277	gi|15924841	Fumarate hydratase	601	*fumC*	SAV1851	Down
A043	gi|15924684	Isocitrate dehydrogenase	845	*citC*	SAV1694	Down
A286	gi|15924412	Glucose-specific enzyme II, PTS system A component	297	*crr*	SAV1422	Down
A287	gi|15924701	Acetate kinase	750	*ackA*	SAV1711	Up
Energy metabolism						
A029	gi|15925597	Malate:quinone oxidoreductase	1353	*mqo2*	SAV2607	Down
A055	gi|15923231	L-lactate dehydrogenase	298	*lctE*	SAV0241	Down
A056	gi|15923246	Xylitol dehydrogenase	78		SAV0256	Down
A063	gi|15924332	Transketolase	684	*tkt*	SAV1342	Down
A071	gi|15925406	Phosphglyceromutase	364	*gpmA*	SAV2416	Down
A183	gi|15924687	Pyruvate kinase	433	*pykA*	SAV1697	Down
A008	gi|15923216	Formate acetyltrasferase	1155	*pflB*	SAV0226	Up
A058	gi|15925514	D-lactate dehydrogenase	972	*Ddh*	SAV2524	Up
A078	gi|15925115	Fructose-bisphosphate aldolase	62	*fbaA*	SAV2125	Up
A281	gi|15923595	Alcohol dehydrogenase	1305	*Adh*	SAV0605	Up
Lipid metabolism						
A027	gi|15924291	Glycerol kinase	1037	*glpK*	SAV1301	Down
A054	gi|15923640	Dihydroxyacetone kinase subunit DhaK	420		SAV0650	Down
A067	gi|15924001	Enoyl-(acyl carrier protein) reductase	526	*fabI*	SAV1011	Down
A072	gi|15923029	Glycerophosphoryl diester phosphodiesterase-like protein	616		SAV0039	Down
A089	gi|15925088	(3R)-hydroxymyristoyl-ACP dehydrogenase	125	*fabZ*	SAV2098	Up
Amino-acid biosynthesis/metabolism						
A039	gi|15923948	NAD-specific glutamate dehydrogenase	970	*gudB*	SAV0958	Down
A046	gi|15923947	Ornithine--oxo-acid transaminase	1027	*rocD*	SAV0957	Down
A045	gi|15925103	Serine hydroxymethyltransferase	365	*glyA*	SAV2113	Down
A049	gi|15924192	Carbamoyl phosphate synthase small subunit	923	*pyrAA*	SAV1202	Up
A051	gi|15924159	Ornithine carbamoyltransferase	556	*argF*	SAV1169	Up
A060	gi|15924160	Carbamate kinase	990	*arcC1*	SAV1170	Up
A280	gi|15924727	Bifunctional 3-deoxy-7-phophoheptulonate sunthase/chorismate mutase	434		SAV1737	Up
Cell division, cell wall, cell envelope biogenesis						
A017	gi|15925144	Glucosamine--fructiose-6-phosphate aminotransferase	167	*glmS*	SAV2154	Down
A032	gi|15925072	UDP-N-acetylmuramoyl-tripeptide--D-alanyl-D-alanine ligase	792	*murF*	SAV2082	Down
A033	gi|15925114	UDP-N-acetylglucosamine-1-carboxylvinyl transferase	1062	*murZ*	SAV2124	Down
A048	gi|15923145	Capsular polysaccharide synthesis enzyme Cap5G	518	*capG*	SAV0155	Down
A274	gi|15924665	Trigger factor	1278	*tig*	SAV1675	Down
Purine and pyrimidine metabolism						
A079	gi|15924058	Phosphoribosylformylglycinamidine synthase	162	*purQ*	SAV1068	Down
A035	gi|15924191	Dihydroorotase	866	*pyrC*	SAV1201	Up
A059	gi|15924190	Aspartate carbamoyltranferase	328	*pyrB*	SAV1200	Up
A080	gi|15925102	Uracil phosphoribosyltransferase	206	*Upp*	SAV2112	Up
Transcription and translation						
A021	gi|15923537	Elongation factor G	411	*fusA*	SAV0547	Down
A031	gi|15923518	Glutamyl-tRNA synthetase	264	*gltX*	SAV0528	Down
A053	gi|15924247	Elongation factor Ts	317	*Tsf*	SAV1257	Down
A061	gi|15924246	30S ribosomal protein S2	465	*rpsB*	SAV1256	Down
A065	gi|15923491	50S ribosomal protein L25	424	*rplY*	SAV0501	Down
A069	gi|15923538	Elongation factor Tu	123	*Tuf*	SAV0548	Down
A070	gi|15925240	50S ribosomal protein L3	516	*rplC*	SAV2250	Down
A076	gi|15924709	30S ribosomal protein S4	532	*rpsD*	SAV1719	Down
A096	gi|15924253	Prolyl-tRNA synthetase	176	*proS*	SAV1263	Down
A108	gi|15925214	DNA-directed RNA polymerase subunit alpha	905	*rpoA*	SAV2224	Down
A276	gi|15924256	Transcription elongation factor NusA	931	*nusA*	SAV1266	Down
A030	gi|15924889	Aspartyl/glutamyl-tRNA amidotrasferase subunit B	336	*gatB*	SAV1899	Up
A081	gi|15922999	Seryl-tRNA synthetase	379	*serS*	SAV0009	Up
A087	gi|15923529	50S ribosomal protein L10	292	*rplJ*	SAV0539	Up
Stress response and heat shock						
A066	gi|15923693	Oxidoreductase	242		SAV0703	Down
A084	gi|15924414	Methionine sulfoxide reductase A	605	*msrA2*	SAV1424	Down
A090	gi|15924413	Methionine sulfoxide reductase B	342	*msrB*	SAV1423	Down
A138	gi|15925129	General stress protein 20U	519	*dps*	SAV2139	Down
A012	gi|15925538	ATP-dependent Clp proteinase chain	2194	*clpL*	SAV2548	Up
Others						
A042	gi|15924396	Tellurite resistance protein	716		SAV1406	Down
A064	gi|15923958	5-oxo-1,2,5-tricarboxilic-3-penten acid decarboxylase	123		SAV0968	Down
A088	gi|15924853	Bacterioferritin comigratory protein	959		SAV1863	Down
A016	gi|15923990	Thimet oligopeptidase-like protein	733		SAV1000	Up
A020	gi|15925280	Urease subunit alpha	862	*ureC*	SAV2290	Up
A044	gi|15923092	Aminoacylase	628		SAV0102	Up
A091	gi|15925281	Urease accessory protein UreE	209	*ureE*	SAV2291	Up
A114	gi|15925318	Dehydrogenase	389		SAV2328	Up
A285	gi|15924697	Metal-dependent hydrolase	609		SAV1707	Up
Unknown function						
A040	gi|15924926	Hypothetical protein	700	-	SAV1936	Down
A062	gi|15923824	Hypothetical protein SAV0834	537	-	SAV0834	Down
A074	gi|15924667	Hypothetical protein SAV1677	436	-	SAV1677	Down
A075	gi|15925559	Hypothetical protein SAV2569	203	-	SAV2569	Down
A083	gi|15924605	Hypothetical protein SAV1615	91	-	SAV1615	Down
A086	gi|15924342	Hypothetical protein SAV1352	256	-	SAV1352	Down
A085	gi|15925677	Hypothetical protein SA2687	746	-	SAV2687	Up

^a^Accession numbers are from the NCBI protein database

^b^Designate ORF number/gene locus in the *S. aureus* Mu50 genome

Interestingly, several proteins associated with lipid metabolism and cell wall synthesis also displayed differential expression. In particular, expression of the glycerol kinase, dihydroxyacetone kinase subunit DhaK, enoyl-(acyl carrier protein) reductase and glycerophophoryl diester phophodiesterase-like protein was lower in the *sav1322* mutant, whereas expression of (3R)-hydroxymyristoyl-ACP dehydrogenase was higher in the *sav1322* mutant when compared with the WT strain. For cell wall synthesis, glucosamine-fructose-6-phophate aminotransferase (GlmS), UDP-N-acetylmuramoyl-tripeptide-D-alanyl-D-alanine ligase (MurF), and UDP-N-acetylglucosamine-1-carboxylvinyl transferase (MurZ) exhibited lower expression levels in the *sav1322* mutant when compared with the WT strain. GlmS is the key enzyme responsible for the synthesis of glucosamine-6-phosphate from fructose-6-phosphate [16], MurF catalyzes the addition of D-Ala-D-Ala to the nucleotide precursor UDP-N-acetylmuramic acid-L-Ala-γ-Glu-meso-diaminopimelate [17], and MurZ catalyzes the condensation of phophoenolpyruvate with UDP-N-acetylglucosamine [18].

Lastly, markedly lower expression levels of proteins involved in the stress response were observed in the *sav1322* mutant, including the general stress protein 20U (Dps), methionine sulfoxide reductase A (MsrA2), and methionine sulfoxide reductase B (MsrB). MsrA2 reduces the S isomer of methionine sulfoxide, and MsrB reduces the R form, providing protection against oxidative stress [19]. In contrast, expression of ClpL, a protein involved in thermotolerance, was up-regulated in the *sav1322* mutant strain.

### In vivo assessment of the sav1322 mutant strain

**Fig. 4:**
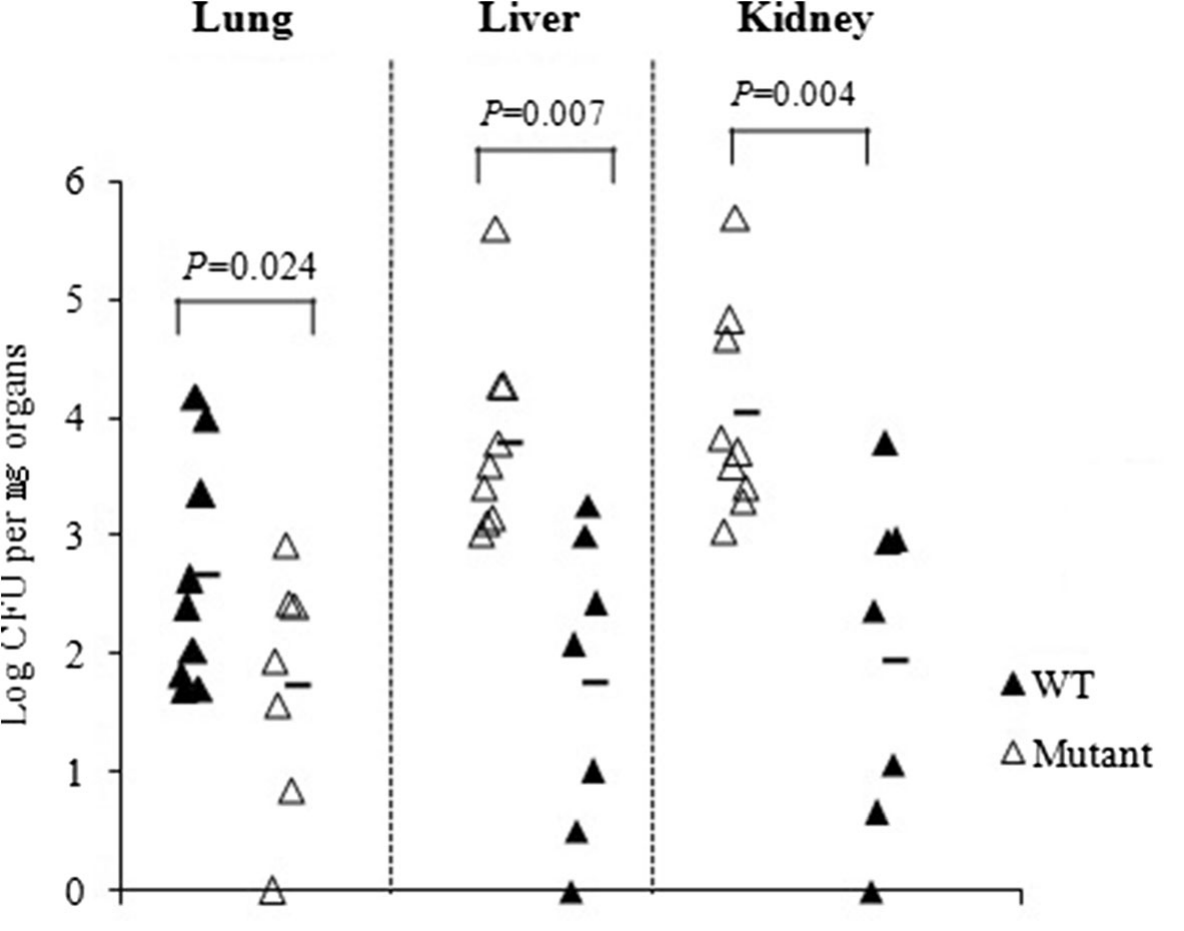
*S. aureus* Mu50 (WT) and *sav1322* mutant pathogenicity. Mice were infected with the WT or *sav1322* mutant strains, and infection progressed for 24 h. CFU (log _CFU per mg organ_) from infected lungs, livers, and kidneys are shown

A mouse model of bacteremia was used to determine how deletion of the SAV1322 locus impacted staphylococcal pathogenicity. Systemic infection following tail vein inoculation of six-week-old female Balb/c mice with the *sav1322* mutant produced a 2-log reduction in bacterial burden found in the lung, liver, and kidney when compared with mice inoculated with the WT strain (Fig. 4).

### Antibiotic susceptibility

**Fig. 5:**
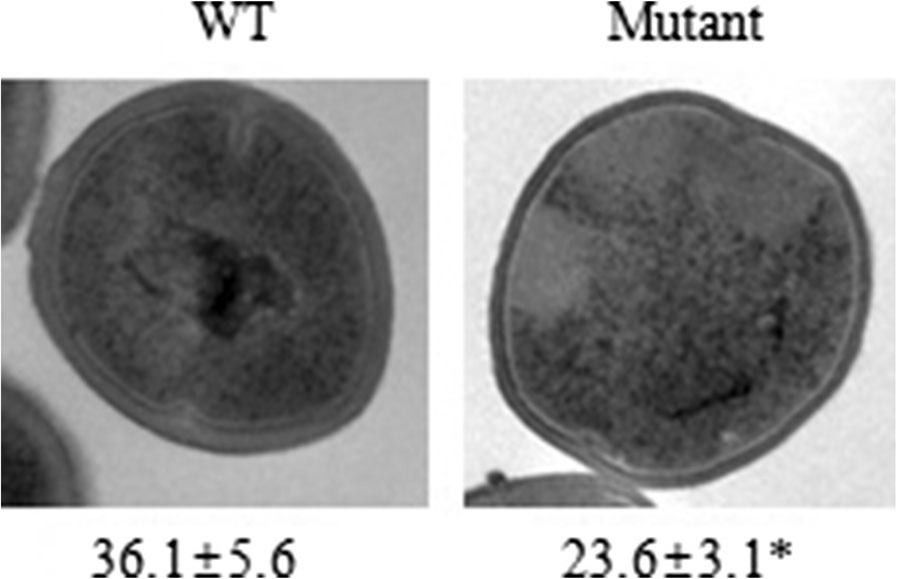
TEM images of representative *S. aureus* Mu50 (WT) and *sav1322* mutant cells. Magnification: 100,000×. Data are expressed as mean ± SD values for cell wall thickness (nm). Asterisks denote statistical significance (*p* < 0.001) when compared with the WT strain

Epsilometer (E)-test strips were used to determine if the *sav1322* mutant strain exhibited altered susceptibility or resistance to several antibiotics. Minimum inhibitory concentrations (MICs) of vancomycin and teicoplanin which target the bacterial cell wall were lower for the *sav1322* mutant when compared to the WT strain (Table 3). We also performed a population analysis to obtain a more detailed evaluation of changes in susceptibility to vancomycin and teicoplanin. Consistent with a lower MIC for both of these antibiotics, we observed a decrease in vancomycin- and teicoplain-resistant subpopulations compared with the WT strain (data not shown). However, those MICs were not recovered in the Com stain. Lower MICs for gentamicin, tigecycline, and tetracycline, which inhibit protein synthesis by binding the 30S subunit of ribosome, were also observed in the *sav1322* mutant strain. The MICs of these antibiotics were fully recovered in Com strain. In addition, the MICs for the bacterial cell membrane-targeting lipopeptide antibiotic, daptomycin, were 3 mg/L and 0.19 mg/L for the WT and *sav1322* mutant strains, respectively. Electron microscopy was used to determine if cell wall thickness was associated with the observed reduction in glycopeptide resistance in the mutant strain. The *sav1322* mutant strain has a thinner cell wall (23.6 ± 3.1 nm) than the WT strain (36.1 ± 5.6 nm) (Fig. 5).

**Table 3:** Antibiotic susceptibility profiles of the WT, *sav1322* mutant, and complementation strains

Antibiotics	MIC (mg/L)		
	WT	*sav1322* mutant	Com
Daptomycin	3	0.19	0.125
Erythromycin	>256	>256	>256
Gentamicin	≥256	1	≥256
Imipenem	>32	1	32
Linezolid	2	2	2
Oxacillin	>256	≥256	≥256
Penicillin	>32	>32	32
Teicoplanin	8	0.5	0.5
Tigecycline	0.5	0.064	0.25
Tetracycline	16	1	8
Vancomycin	8	0.75	0.75

## Discussion

The SAV1321/SAV1322 TCRS system is a histidine kinase sensor/response regulator in the wild-type *S. aureus* Mu50. However, the functions of SAV1321/SAV1322 TCRS-associated genes are not yet known. With this study, we elucidate some of these functions by examining phenotypic, transcriptomic, and proteomic changes in a *sav1322* mutant strain in response to different environmental stressors.

Heat-shock proteins (Hsps) are essential for stress tolerance and survival under protein-denaturing conditions. Four Hsp classes have been identified in Gram-positive bacteria. Class I Hsps comprise classical Hsps that are encoded by genes within the groESL and dnaK operons, and a cytoplasmic repressor HrcA controls expression of these proteins. Sig B controls Class II Hsps, while class II Hsps are Clp proteases that are typically controlled by the transcriptional repressor CtsR. Class IV Hsps are encoded by genes that are not controlled by HrcA, SigB, or CtsR [3, 20]. Interestingly, our data suggest that the *sav1322* mutant strain is more sensitive to heat and cold than the WT strain. Microarray data further demonstrate that the transcription of class I Hsp and III Hsp genes. More specifically, *hrcA*, *ctsR*, *dnaJ*, *dnaK*, and *grpE* transcript levels are higher in the *sav1322* mutant. However, expression of cold-shock genes (*cspA* and *cspB*) is not affected in the mutant. These results suggest that SAV1322 may play a role in temperature adaptation by regulating the expression of chaperone proteins. While CtsR represses the transcription of genes encoding class I and class III Hsps, however, we noted that expression of the class III chaperone genes *clpC* and *clpB* is higher in the *sav1322* mutant strain.

The clpC operon in *S. aureus* comprises four genes denoted *ctsR*, *mcsA*, *mcsB*, and *clpC*. One previous study has reported that a mutation in *mcsB* results in hypersensitivity to heavy metal, temperature, osmotic pressure, and oxidative stresses [21]. ClpC proteases are implicated in stress resistance, and *clpC* and *clpB* transcription increases during acid shock in *S. aureus* [22]. In this study, the *sav1322* mutant exhibited greater resistance to oxidative stress. This may be partially explained by the higher levels of *clpC* transcription observed in this strain.

Our proteomic data further revealed that the expression of 72 proteins is altered at least two-fold in the *sav1322* mutant when compared with the WT strain. Many of these proteins are directly or indirectly involved in important cellular processes, including the TCA cycle. The TCA cycle have been implicated in antibiotic resistance, and, possibly, staphylococcal virulence [23, 24]. Inactivation of the TCA cycle delays the resolution of cutaneous ulcers in soft tissue infections of the mouse [25], and disruption of *citZ* (citrate synthase), *citC* (isocitrate dehydrogenase), and *citB* (aconitate hydratase) genes prevents bacterial capsule formation [26]. Surmann et al. analyzed survival and physiological adaptation of *S. aureus* after internalization by human lung epitheilial cell lines A549. A number of TCA cycle enzymes including CitZ, CitC, SucC and SdhA increased in level upon internalization by this epithelial cell. Moreover, L-lactate dehydrogenase (LctE) increased in level after internalization by A549 and human embryonic kidney cells HEK 293, indicating the supplementary utilization of fermentative enzymes under microaerobic conditions [27]. Our proteomic results demonstrate that expression of proteins involved in TCA cycle, such as OdhB, CitC, PdhD, and FumC, is lower in the *sav1322* mutant. Furthermore, several major fermentative and anaplerotic pathway components were also decreased in the mutant, in particular LctE, malate:quinone oxidoreductase (Mqo2) and pyruvate kinase (PykA). These results suggest that the down-regulation of energy-providing pathways may be energetically less efficient in the mutant compared with the wild type strain, then the recovered CFU were lower in tissue of mice infected with the mutant (Fig. 4).

Furthermore, LctE catalyzes the conversion from pyruvate to lactate. The L-lactate produced by this process allows *S. aureus* to maintain redox homeostasis during nitrosative stress caused by activated phagocytes and is essential for virulence [28]. In this study, the protein level of LctE was decreased in mutant compared with WT strain. This may resulted in decrease the production of L-lactate in the mutant and cannot survive from the attack of the host, including the radial nitric oxide.

Proteins involved in protein synthesis are also altered upon deletion of SAV1322 including 30S ribosomal protein S2, 30S ribosomal protein S4, and several aminoacyl-tRNA synthetase. In accordance with this proteomic result, the lower MICs of tetracycline, tigecycline, and gentamicin that bind 30S subunit of ribosome were observed in the *sav1322* mutant, and fully restored in Com strain. These results suggest that SAV1322 may play a role in antibiotic resistance to tetracycline, tigecycline, and gentamicin, but further studies are needed to elucidate the underlying specific mechanism.

Proteins involved in cell wall biosynthesis are also altered upon deletion of SAV1322. Cell wall synthesis is crucial for bacterial division and growth, and it is an important target for antibiotics, including penicillin, vancomycin, and teicoplanin. As the number of reported MRSA strains increases, vancomycin has become the first-line treatment for staphylococcal infections. However, the use of vancomycin has led to the emergence of VISA strains [1]. Typically, VISA exhibits thick cell walls and reduced autolysis [1, 12]. We speculate that the altered expression of cell wall metabolism-related proteins in the *sav1322* mutant results in lower MICs for several antibiotics that inhibit cell wall synthesis. Moreover, the *sav1322* mutant had thinner cell walls and is more susceptible to Triton X-100-induced autolysis and lysostaphin-induced cell lysis. Curiously, we have observed that a susceptibility of vancomycin and teicoplanin attenuated by deletion of SAV1322 cannot be readily complemented. Explanations for this phenomenon could be due to: (i) incomplete recovery of the cell wall thickness, (ii) the highly complex glycopeotide resistance regulatory cross-talk in VISA or (iii) the involvement of posttranscriptional mechanisms, as we detected by comparing transciptomic and proteomic dtata, and thus will require further study.

In this study, it was observed that the lack of correlation between transcript level and identified proteins. There is a remarkable series of processes between transcription and translation, including spanning the transcription, processing and degradation of mRNAs to the translation, localization, modification and programmed destruction of proteins. The abundances of protein reflect a dynamic balance among these processes [29]. Other factors include the protein stability. The half-life of different proteins can vary from minutes to days, whereas the degradation rate of mRNA would fall within a much tighter range (several hours). Another factor might be of solubility of proteins in the buffers used in this study. Some highly expressed genes probably encode for highly insoluble proteins that are difficult to analyze even in the presence of detergents.

## Conclusions

This study is the first to provide a functional transcriptomic and proteomic analysis of the response regulator SAV1322 in *S. aureus*. SAV1322 plays a pronounced role in temperature adaptation, resistance to antibiotics, and virulence. In addition, it influences the expression of a large number of genes involved in the heat shock response, cell wall metabolism, energy metabolism, and response to other environmental stressors. Our findings provide valuable insight into antibiotics resistance and staphylococcal pathogenicity. Both may help shape future studies on antibiotic resistance trends and optimal antibacterial treatment strategies.

## Methods

### Construction of the sav1322 mutant and complementation strains

The *S. aureus* Mu50 was used to generate a SAV1322 knockout mutant strain using an allelic exchange method described previously [30]. Briefly, sequences flanking SAV1322 were amplified by PCR using primers that contained attB1 (5′-GGGGACAAGTTTGTACAAAAAAGCAGGCT-) and attB2 (5′-GGGGACCACTTTGTACAAGAAAGCTGGGT-) sites on the respective up- and downstream target sequences. PCR fragments were cloned into the pKOR1 vector, and then introduced into *S. aureus* Mu50 by electroporation. To select for single-crossover mutants, electroporated clones were cultured overnight at 43 °C. Single-crossover mutants were selected and cultured in antibiotic-free broth medium to facilitate plasmid excision, then subjected to anhydrotetracycline induction to select a non-plasmid-carrying mutant. Successful deletion of SAV1322 was verified by PCR using the specific primers covering target sequences (UP FWD and DOWN REV) and sequence analysis. For the complementation strain, a 1620-bp fragment of the SAV1322 gene was PCR-amplified using Mu50 genomic DNA as a template with the primer sets Com-F and Com-R (Fig. 1a), then cloned into the pYT3 vector and introduced into the *sav1322* mutant strain as described previously [31]. To confirm of complementation, reverse transcription-PCR analysis was performed. Total RNA was isolated from the WT, mutant, and complementation strains and reverse-transcribed using random hexamers and Moloney murine leukemia virus reverse transcriptase for 60 min at 42 °C. Resulting cDNA fragments were used as templates for PCR amplification of the target gene.

### Stress response analyses

Strains were cultured overnight in tryptic soy broth (TSB) at 37 °C and diluted to an OD_600_ reading of 0.3. Cells were then incubated at 25 and 46 °C with shaking, while OD_600_ readings were taken at 1 h intervals for 7 h. To investigate the oxidative stress response, WT and *sav1322* mutant cultures grown overnight were plated on tryptic soy agar (TSA). A disk containing 10 μl H_2_O_2_ (15 % or 30 %) was placed on the prepared agar plates, then incubated at 37 °C for 18 h.

### Microarray analysis

RNA extraction, cDNA labeling, hybridization, and microarray data analyses were carried out according to protocols described previously [32]. Cells were grown exponentially to an OD_600_ of approximately 0.5, then harvested. A customized *S. aureus* high-density synthetic oligonucleotide array was designed using 982 predicted open reading frames (ORF) with the GenBank accession number NC_002745 (NimbleGen Systems Inc., Madison, WI, US). The ORF listed in Additional file 3. Arrays were scanned with a NimbleGen MS 200 Microarray scanner set at 532 nm with a resolution of 2 μm to produce images (TIFF format) according to the manufacturer’s instructions. Array data export processing and analysis was performed using NimbleScan v2.5 (Gene Expression RMA algorithm). Adjustments for batch effects were made with data-to-filter non-biological experimental variation (http://biosun1.harvard.edu/complab/batch/). A single raw intensity value was determined for each gene in every array by averaging spot replicates of all probes for each of the genes. Gene signal values were log2 transformed. Statistical significance of the expression data was determined using the local-pooled-error test and fold change in which the null hypothesis was that no difference exists between two groups. Adjusting *P*-values with a Benjamini-Hochberg algorithm controlled the false discovery rate. Hierarchical cluster analysis was performed using complete linkage and Euclidean distance as a measure of similarity.

### Two-dimensional gel electrophoresis and protein identification

Cell extracts from overnight WT and *sav1322* mutant cultures were separated by two-dimensional gel electrophoresis. Aliquots containing approximately 800-μg protein were diluted in 2D-PAGE rehydration buffer (7 M urea, 2 M thiourea, 4 % CHAPS, 0.4 % DTT) to a final volume of 350 μl and centrifuged at 100,000 g for 30 min to remove insoluble material. For the first dimension, samples were run on pH 4–7 IPG strips (GE Healthcare Life Sciences, UK) on a Multiphor apparatus II instrument (Amersham Biosciences, UK) according to the manufacturer’s instructions. Strips were incubated in equilibration buffer (6 M urea, 20 mM Tris-HCl, pH 8.8, 2 % SDS, 20 % glycerol, 2.5 % acrylamide, and 5 mM tributyl phosphine) for 20 min. The second dimension separation was performed using 8–16 % linear gradient SDS-polyacrylamide gels. Gels were stained with colloidal Coomassie solution (ProteomeTech, Seoul, Korea) according to the manufacturer’s instructions. Differences in the expression profiles of spots were quantified using the ImageMasterTM 2D Platinum software (Amersham Biosciences, UK). In-gel digestion of the protein spots was performed as described previously [33]. Resulting tryptic peptides were analyzed using reversed phase capillary HPLC directly coupled to a Finnigan LCQ ion-trap mass spectrometer (LC-MS/MS) previously described [34]. Individual spectra from MS/MS were processed using TurboSEQUEST software (ThermoQuest, San Jose, CA). The generated peak list files were used to query the MSDB database or NCBI using the MASCOT program (http://www.matrixscience.com).

### Antimicrobial susceptibility

MICs for different antimicrobial agents were determined with E-test strips (AB bioMérieux, Marcy I’Etoile, France). A sterile cotton swab was immersed in each bacterial culture grown to a 0.5 McFarland turbidity standard and streaked on Mueller-Hinton agar (Difco, Detroit, MI) plates. Plates were incubated at 37 °C, and MICs were measured after a 24-h incubation period according to the manufacturer’s instructions.

### Transmission electron microscopy (TEM)

WT and *sav1322* mutant cells were prepared and visualized by TEM as described previously [35]. Morphometric evaluation of cell wall thickness was determined using photographs at 20,000× magnification. At least 30 cells from each strain with almost equatorial cut surfaces were measured and results are expressed as mean ± SD values. The statistical significance of cell wall thickness was evaluated by the Student’s *t*-test (*p* < 0.001).

### Mouse infection model

All animal experiments were conducted in accordance with guidelines and the approval of the Institutional Animal Care and Use Committee of Korea Centers for Disease Control and prevention (KCDC-030-12-2A). WT and *sav1322* mutant strains were grown and harvested at the mid-point of the exponential growth phase, washed in sterile PBS, and resuspended in PBS to a concentration of 1 × 10^8^ cells per 0.1 ml. Female Balb/c mice (aged 5–6 weeks) were inoculated with this suspension or PBS via the tail vein. The experiment was performed with ten mice for each *S. aureus* strain and five mice for the PBS control. Mice were euthanized 24 h after infection, and lungs, livers, and kidneys were removed and homogenized in sterile PBS. Homogenates were diluted in PBS and plated on TSA, then incubated overnight in 37 °C. The bacterial burden in each organ was assessed by CFU counts. The student’s *t*-test was used to determine statistical significance between samples.

## Additional files

Additional file 1: Colony forming units (CFU) of the *S. aureus* Mu50 (WT), sav1322 mutant, and complementation (Com) strains at 37 °C, 46 °C, 25 °C. (DOCX 22 kb) Additional file 2: 2-D PAGE of the *S. aureus* Mu50 (WT) and *sav1322* mutant strains. (DOCX 568 kb) Additional file 3: List of probes for microarray. (XLSX 58 kb)

## Abbreviations

CFU: Colony-forming unit
MRSA: Methicillin-resistant *S. aureus*
ORF: Open reading frame
PBS: Phosphate-buffered saline
TCRS: Two-component regulatory system
TSA: Tryptic soy agar
TSB: Tryptic soy broth
VISA: Vancomycin-intermediate *S. aureus*
VRSA: Vancomycin-resistant *S. aureus*
