# Supplementary material for: The SAV1322 gene from Staphylococcus aureus: genomic and proteomic approaches to identification and characterization of gene function
Source: BMC Microbiol. 2016 Sep 6;16(1):206. doi: 10.1186/s12866-016-0824-2 (PMC5013637; doi:10.1186/s12866-016-0824-2)
Supplement: Additional file 1: — Colony forming units (CFU) of the S. aureus Mu50 (WT), sav1322 mutant, and complementation (Com) strains at 37 °C, 46 °C, 25 °C. (DOCX 22 kb) [file 12866_2016_824_MOESM1_ESM.docx]

**Figure S1.** Colony forming units (CFU) of the *S. aureus* Mu50 (WT, filled triangle), *sav1322* mutant (open triangle), and complementation (Com, filled square) strains at (a) 37 °C, (b) 46 °C, and (c) 25 °C. Data are expressed as mean ± SD values for the measurements.
