# Supplementary material for: The SAV1322 gene from Staphylococcus aureus: genomic and proteomic approaches to identification and characterization of gene function
Source: BMC Microbiol. 2016 Sep 6;16(1):206. doi: 10.1186/s12866-016-0824-2 (PMC5013637; doi:10.1186/s12866-016-0824-2)
Supplement: Additional file 2: — 2-D PAGE of the S. aureus Mu50 (WT) and sav1322 mutant strains. (DOCX 568 kb) [file 12866_2016_824_MOESM2_ESM.docx]

(a)


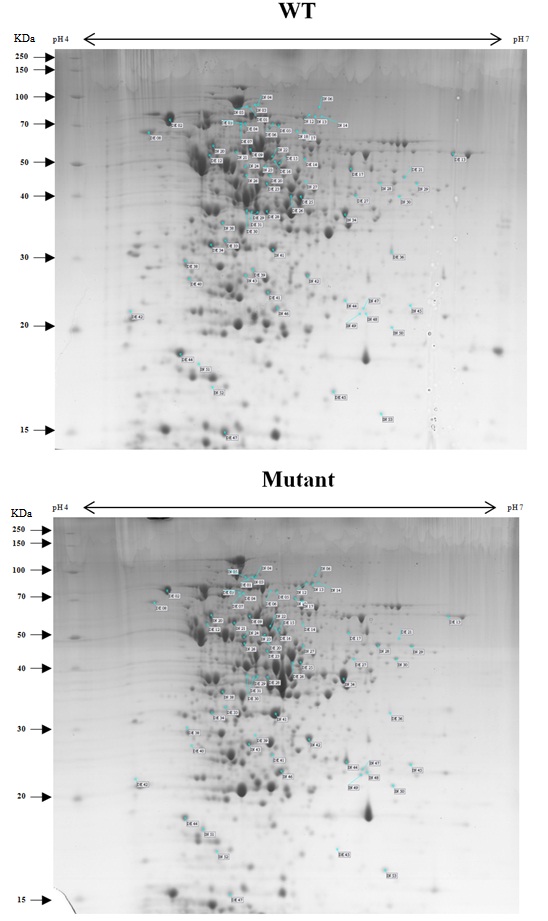


(b)
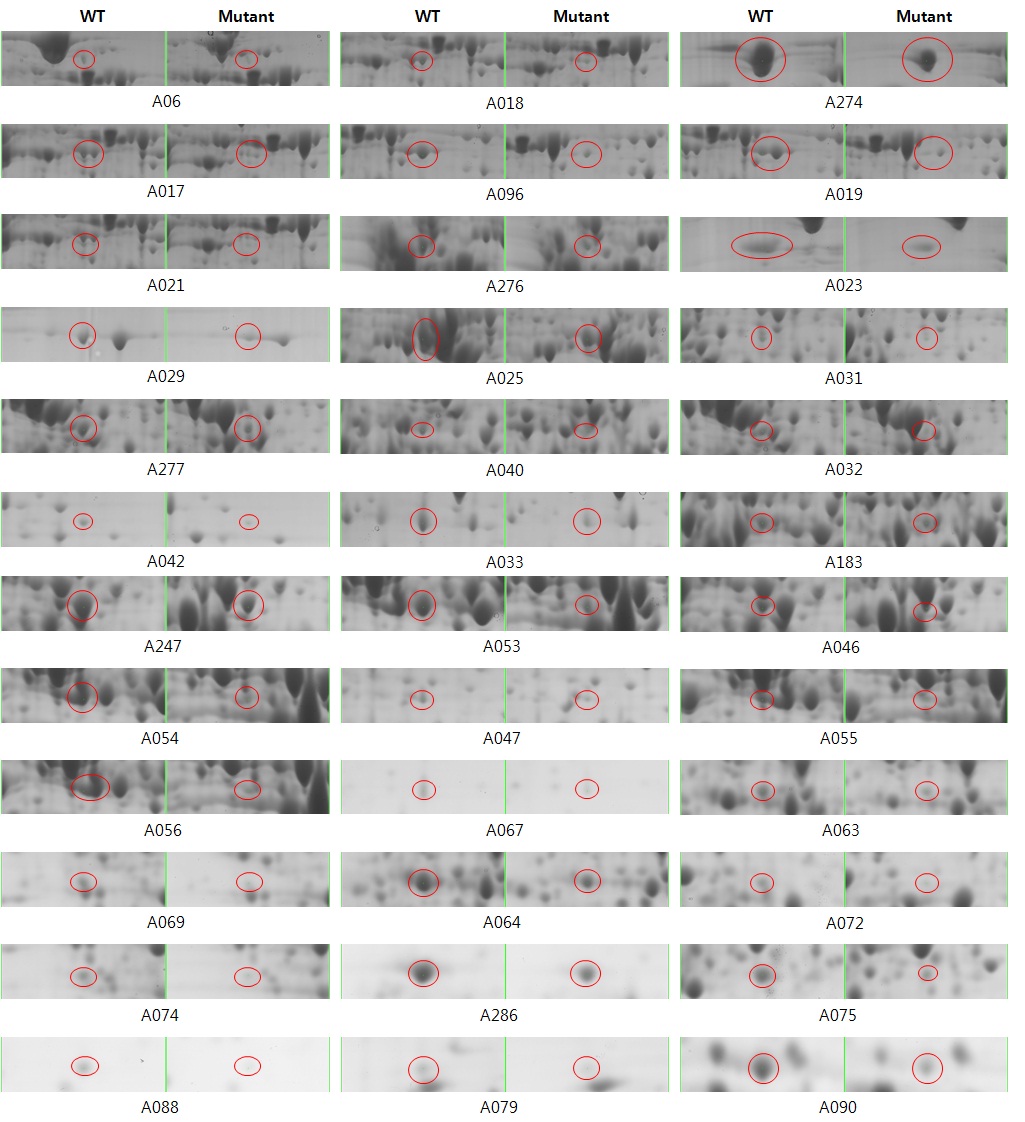


(c)


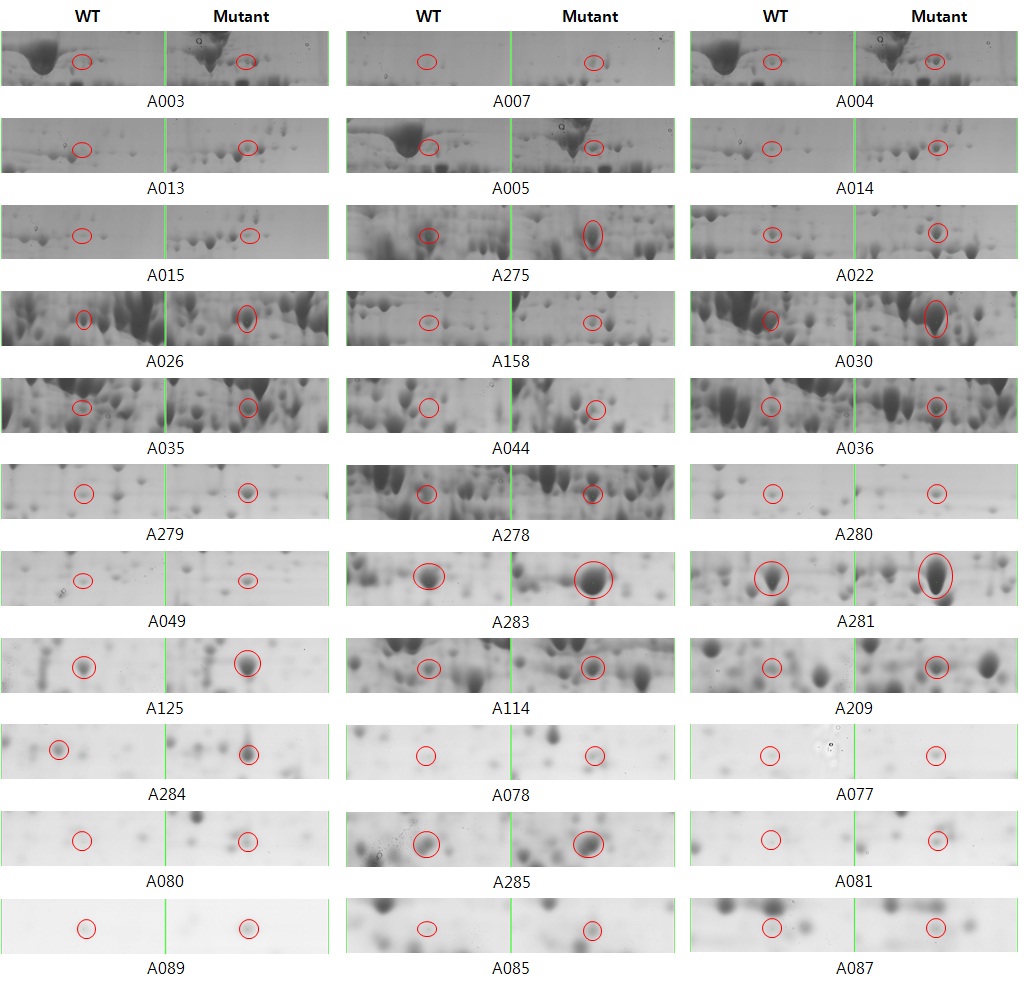


**Figure S2.** 2-D PAGE profiles of the *S. aureus* Mu50 (WT) and *sav1322* mutant strains (a). The protein extracts (800-µg) was separated on 2D gels by using immobilized pH gradient strips in the pH range of 4-7. Gels were stained with colloidal Coomassie Brilliant Blue. Differences in the expression profiles of spots were quantified using the ImageMasterTM 2D Platinum software. Close-up images of decreased (b) and increased (c) expression of the protein spots.
